# P-292. Impact of Emergency Department Pharmacist-Led HIV Pre-Exposure Prophylaxis Initiation in Patients with Positive Sexually Transmitted Infection Testing

**DOI:** 10.1093/ofid/ofaf695.513

**Published:** 2026-01-11

**Authors:** Bushra Altabbaa, Kate Shupp, Lewis Hunter Reese, Ann A Avery, Morgan K Morelli

**Affiliations:** MetroHealth Medical Center, Westlake, OH; MetroHealth Medical Center, Westlake, OH; MetroHealth Medical Center, Westlake, OH; Case Western Reserve University School of Medicine, Cleveland, Ohio; MetroHealth Medical Center, Westlake, OH

## Abstract

**Background:**

Approximately 30,000 people in the U.S. are diagnosed with HIV annually. Despite pre-exposure prophylaxis (PrEP) —medication to prevent HIV acquisition—proven to be 99% efficacious in preventing sexual transmission, nearly 80% of eligible Americans are not prescribed it, and fewer than 10% with an indication are aware of its existence. The CDC recommends PrEP in individuals diagnosed with a sexually transmitted infection (STI) in the past six months. The emergency department (ED) is a common site for STI care. Clinical pharmacists in our ED review STI results and ensure proper treatment is received utilizing a collaborative practice agreement to contact patients and initiate therapy. This study leverages that workflow to evaluate a novel approach to increase PrEP prescribing among this group.

Baseline Characteristics

**Figure:**
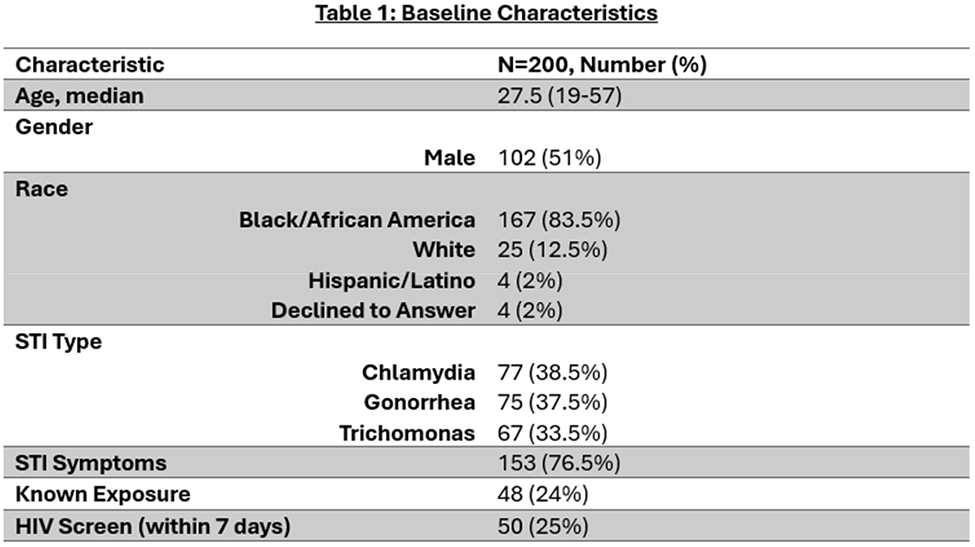

**Methods:**

This was a retrospective quality improvement study of adult patients who tested positive for an STI in 4 of MetroHealth emergency departments and were contacted to discuss PrEP initiation from 9/5/24 through 3/1/25. Exclusions included known HIV positive, age < 18, pregnancy, or hospitalization. A pharmacist could order and review labs and initiate PrEP. Patients were linked to primary care for ongoing management. The primary outcome was the number of patients started on PrEP. Secondary outcomes included the number of patients who were reached, agreed to labs, and completed labs.

**Results:**

200 patients were included. Recent HIV screening was obtained in only 25% of patients (all negative). Pharmacists successfully contacted 137 (68.5%), with 67 (48.9%) agreeing to PrEP. All were agreeable to labs, but only 10 (14.9%) obtained them. Three patients were prescribed Emtricitabine/tenofovir disoproxil fumarate and 1 started long acting (LA)- Cabotegravir. Two additional patients interested in LA-Cab did not follow up.

**Conclusion:**

Pharmacist-led PrEP initiation in the ED successfully identified and contacted individuals at risk of HIV. While most patients were reached and half expressed interest in PrEP, only a few completed necessary labs and were ultimately prescribed PrEP. In general, patients were receptive to the calls and appreciated the information and follow up. Initiating PrEP in the ED offers a critical opportunity to provide HIV prevention to this high-risk population.

**Disclosures:**

All Authors: No reported disclosures

